# Real-world anti-amyloid therapy beyond traditional symptomatic populations: a longitudinal case series

**DOI:** 10.3389/fpsyt.2026.1900651

**Published:** 2026-08-28

**Authors:** Jayoung Han, Yuan Fang, Darlingtina Esiaka, Olufunmilola Abraham

**Affiliations:** 1 Department of Pharmaceutical Sciences, Fairleigh Dickinson University College of Pharmacy and Health Sciences, Florham Park, NJ, United States; 2 Department of Mathematics & Statistics, Old Dominion University College of Sciences, Norfolk, VA, United States; 3 Center for Health, Engagement and Transformation and Department of Behavioral Science, University of Kentucky College of Medicine, Lexington, KY, United States; 4 Department Chair & Professor, U.S. Surgical Endowed Professor in Pharmacy Administration, Department of Pharmacy Practice & Science, University of Kentucky College of Pharmacy, Lexington, KY, United States

**Keywords:** Alzheimer’s disease, anti-amyloid therapy, biomarkers, donanemab, lecanemab, longitudinal trajectories, preclinical stage, real-world evidence

## Abstract

**Introduction:**

Anti-amyloid monoclonal antibodies are approved as disease-modifying therapies for early Alzheimer’s disease (AD), but real-world evidence remains limited, particularly among individuals treated during preclinical or minimally symptomatic stages. This study characterized clinical and biomarker trajectories among anti-amyloid therapy recipients across disease stages.

**Method:**

We conducted a longitudinal retrospective case series of three biomarker-positive individuals from the Alzheimer’s Disease Neuroimaging Initiative who received lecanemab or donanemab. Clinical, cognitive, cerebrospinal fluid, plasma biomarker, and amyloid PET data were examined.

**Results:**

In all three cases, most of the observation period preceded anti-amyloid therapy initiation. Clinical trajectories varied by biomarker profile and treatment timing. Two participants received donanemab while cognitively normal or at an early transitional stage despite persistent amyloid- and tau-positive profiles. One participant received lecanemab after clinically significant impairment emerged. Cases 1 and 2 showed prolonged preservation of cognitive and functional performance despite sustained AD biomarker abnormalities and amyloid PET positivity. In case 2, age- and education-adjusted Wechsler Logical Memory testing detected progression to mild cognitive impairment despite preserved Mini-Mental State Examination and Clinical Dementia Rating scores. In contrast, case 3 showed persistent cognitive impairment and progression toward mild dementia. Conclusion: This case series illustrates substantial heterogeneity among real-world anti-amyloid therapy recipients. Persistent biomarker abnormalities may precede clinical decline for years, especially among highly educated individuals with substantial cognitive reserve. Conventional global screening tools may underestimate early decline and affect treatment eligibility decisions. Although not generalizable, these observations underscore the need for larger real-world studies characterizing clinical trajectories following anti-amyloid therapy.

## Introduction

Alzheimer’s disease (AD) is a progressive neurodegenerative disorder characterized by gradual decline in memory, cognition, and daily functioning (1). In the United States, the number of individuals living with AD is expected to continue rising substantially with population aging, creating increasing clinical and societal burden (1, 2). Current models conceptualize AD as a biological continuum beginning years before overt cognitive symptoms become clinically apparent (3). During this prolonged preclinical phase, pathological accumulation of amyloid beta and tau may occur despite preserved global cognitive performance (3, 4).

Recent therapeutic advances have introduced anti-amyloid monoclonal antibodies, including lecanemab and donanemab, as disease-modifying treatments for early AD. The U.S. Food and Drug Administration approved lecanemab on July 6, 2023, and donanemab on July 2, 2024, based on reductions in amyloid plaque burden and clinical benefits demonstrated by slowing both cognitive and functional decline (5, 6). However, most available evidence remains derived from highly selected clinical trial populations (7). A key unresolved issue is how biomarker-defined disease states align with clinical presentation in real-world treatment contexts, particularly when therapies are initiated outside trial-defined symptomatic stages. Recent longitudinal evidence suggests that AD biomarkers become abnormal many years before overt cognitive impairment, with amyloid abnormalities preceding measurable cognitive decline by more than a decade (8). Consequently, an increasing number of biomarker-positive individuals may be considered for anti-amyloid therapy while remaining clinically unimpaired or minimally symptomatic, yet little is known about their longitudinal clinical trajectories following treatment initiation. As a result, relatively little is known regarding how these therapies are being used in real-world clinical settings, particularly among individuals treated outside conventional trial-defined symptomatic populations.

Emerging real-world studies of lecanemab have primarily focused on utilization patterns and patient characteristics using administrative or claims-based datasets. However, these datasets generally lack detailed longitudinal cognitive and biomarker information and are typically restricted to patients meeting approved clinical inclusion criteria, providing limited insight into individuals treated during preclinical or minimally symptomatic stages of disease (9–11). Furthermore, longitudinal real-world evidence describing patient-level trajectories following donanemab initiation remains extremely limited.

To address this gap, the present case series characterizes longitudinal clinical, cognitive, and biomarker trajectories across three distinct clinical scenarios spanning the AD continuum. The purpose of this case series was to characterize heterogeneous clinical, cognitive, and biomarker trajectories among individuals receiving lecanemab or donanemab, including patients treated during preclinical stages of AD. By integrating longitudinal biomarker profiles, imaging findings, and repeated clinical assessments, this study aims to provide clinically detailed insight into disease progression patterns in the evolving anti-amyloid treatment era.

## Method

### Data source and case selection

This retrospective longitudinal case series included three biomarker-positive individuals identified from the Alzheimer’s Disease Neuroimaging Initiative (ADNI) who received anti-amyloid monoclonal antibody therapy with lecanemab or donanemab. ADNI is a multicenter longitudinal observational study launched in 2004 to develop and validate clinical, imaging, genetic, and fluid biomarkers for Alzheimer’s disease (AD) progression. ADNI enrolls cognitively unimpaired individuals, participants with mild cognitive impairment, and individuals with AD dementia, with standardized collection of clinical, neuropsychological, neuroimaging, and biospecimen data across participating sites. Of the 23 patients who had received anti-amyloid therapies at the time of analysis (March 2026), three with available longitudinal follow-up data were included in this case series. The remaining 20 patients had been enrolled in ADNI between 2023 and 2025 and did not yet have sufficient longitudinal follow-up for inclusion. For the current study, cases were selected based on (1) confirmed amyloid and tau biomarker positivity, (2) documented exposure to anti-amyloid therapy, and (3) availability of longitudinal clinical, cognitive, and biomarker data before and after therapy initiation. All participants in ADNI provided informed consent, and study procedures were approved by local institutional review boards.

### Clinical, biomarker, and imaging data

Longitudinal clinical, cognitive, and functional data were reviewed for each case to characterize individual disease and treatment trajectories across the AD continuum. Clinical status (cognitively normal, mild cognitive impairment (MCI), mild dementia) was defined using ADNI diagnostic criteria based on Mini-mental State Examination (MMSE), Clinical Dementia Rating (CDR), and neuropsychological assessment results. Available cerebrospinal fluid (CSF) and plasma biomarkers reflecting amyloid pathology, tau pathology, and neurodegeneration were examined using standardized ADNI acquisition protocols. Amyloid positron emission tomography (PET) imaging studies were evaluated longitudinally to assess changes in cerebral amyloid burden over time. Amyloid PET status (elevated vs. non-elevated) was based on the ADNI-derived classification provided in the study database, with variable definitions and coding obtained from the ADNI Data Dictionary available through the ADNI Study Files portal (12).

### Data analysis

Given the small sample and case-series design, analyses were descriptive, with emphasis on within-case longitudinal patterns rather than cross-case statistical comparison. Findings were summarized qualitatively, with emphasis on longitudinal changes in clinical presentation, cognitive performance, functional status, fluid biomarkers, and amyloid PET imaging following anti-amyloid therapy exposure. Clinical and biomarker findings were analyzed within each case and compared descriptively across cases to identify heterogeneity in treatment response and disease progression.

## Results

### Case descriptions

**Figure 1:**
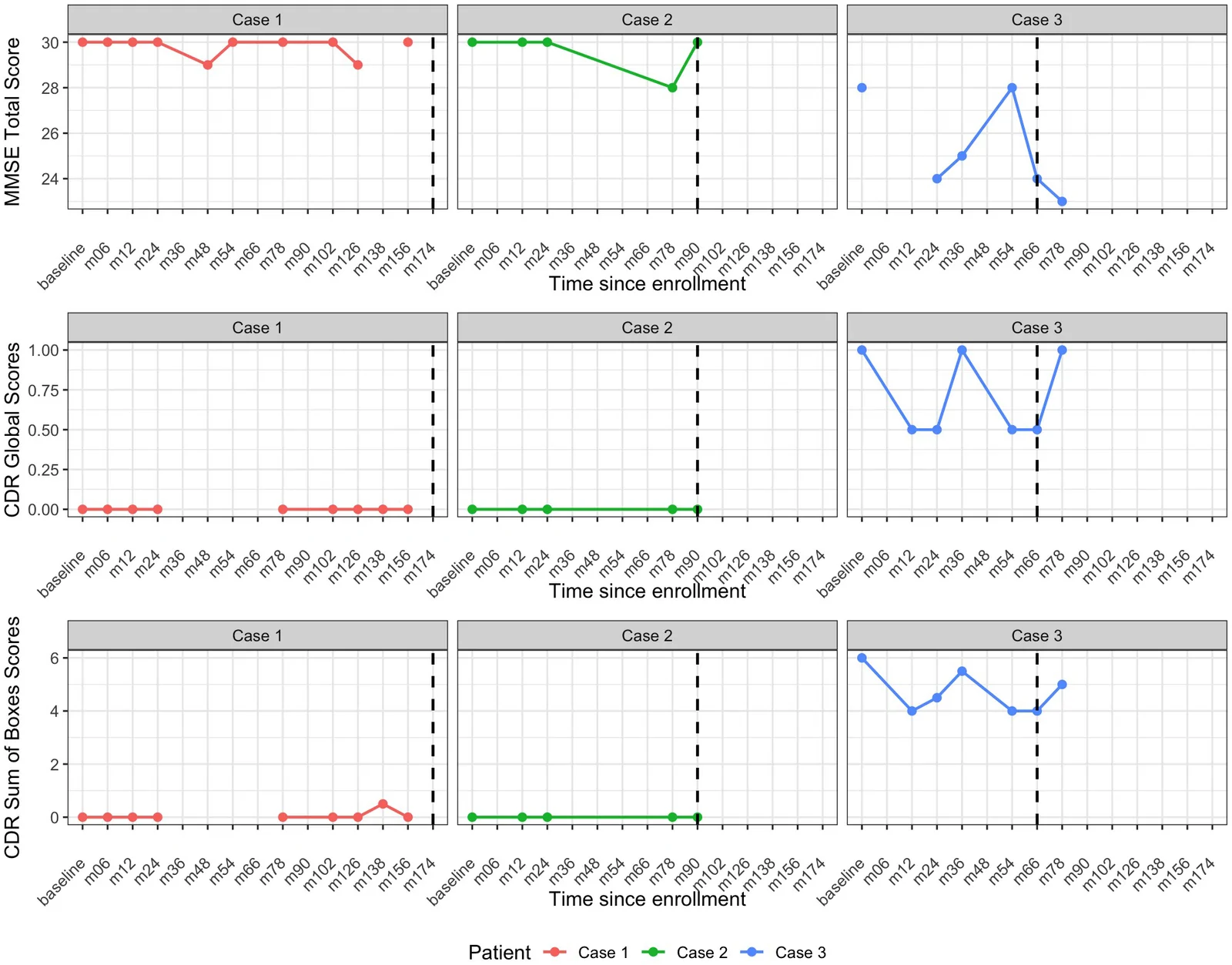
Longitudinal trajectories of cognitive assessments across three cases. MMSE, Mini-Mental State Examination; CDR, Clinical Dementia Rating; CDR-SB, Clinical Dementia Rating Sum of Boxes. Time is shown as months since ADNI enrollment. Baseline represents the first available ADNI visit. Data points represent available observations; gaps reflect missing or unavailable assessments. Higher MMSE scores indicate better cognitive performance, whereas higher CDR Global and CDR-SB scores indicate greater clinical impairment. The vertical dashed line in each panel marks the visit at which anti-amyloid therapy was first documented (case 1, month 174; case 2, month 90; case 3, month 66).

**Figure 2:**
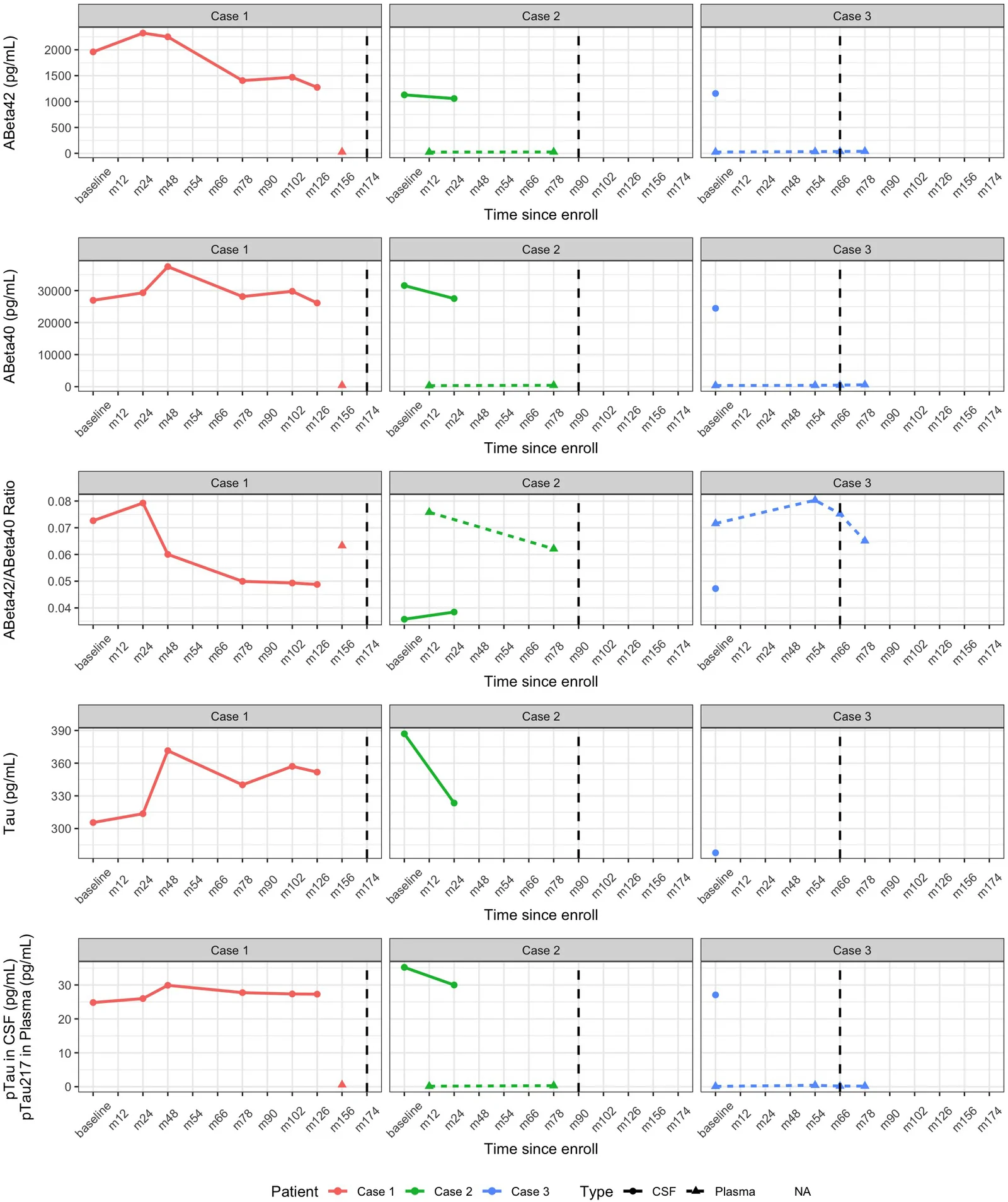
Longitudinal biomarker trajectories across three cases. Aβ42, amyloid beta 42; Aβ40, amyloid beta 40; pTau, phosphorylated tau; CSF, cerebrospinal fluid. Time is shown as months since ADNI enrollment. Baseline represents the first available ADNI visit. Data points reflect available observations only; gaps indicate unavailable biomarker assessments at that visit. The vertical dashed line in each panel marks the visit at which anti-amyloid therapy was first documented (case 1, month 174; case 2, month 90; case 3, month 66).

Longitudinal cognitive characteristics of the three cases are presented in **Figure 1**, whereas longitudinal cerebrospinal fluid and plasma biomarker findings are presented in **Figure 2**. Clinical and functional characteristics are summarized in **Table 1**. Time is expressed as months since ADNI enrollment, and the visit at which anti-amyloid therapy was first documented is stated for each case.

**Table 1:** Longitudinal clinical and functional characteristics of the three cases.

Characteristics	Case 1	Case 2	Case 3
Clinical trajectory	Cognitively normal throughout follow-up	Cognitively normal progressed to MCI	Persistent MCI progressing to mild dementia
Final clinical status	Cognitively normal despite biomarker positivity	MCI with high diagnostic confidence	Mild dementia
Anti-amyloid therapy	Donanemab (month 174, September 2025; no posttreatment follow-up)	Donanemab (February 2026, immediately before the month 90 visit; <1 month)	Lecanemab (month 66, January 2025; ~12 months, through month 78)
Follow-up duration	~15 years	~7.5 years	~6.5 years
Amyloid PET findings	Elevated cortical amyloid deposition across regions 1–5	Elevated cortical amyloid deposition across regions 1–5	Persistent amyloid elevation, primarily region 1
Subjective memory complaints	Not reported	Present at m90	Persistent throughout follow-up
Caregiver-reported memory concerns	Not reported	Present at m90	Persistent throughout follow-up
Objective memory impairment	No	Present at m90 (logical memory II)	Persistent throughout follow-up
FAQ trajectory	0 throughout except transient score of 2 at m12	Preserved functional status	Progressive functional decline
Biomarker interpretation	Transition from borderline profile to sustained A+T+ AD profile	Persistent and progressively worsening AD pathology	Sustained AD pathological progression
Clinical–biomarker dissociation	Marked biomarker positivity despite preserved cognition	Significant biomarker burden preceding clinical impairment	Concordant biomarker and clinical progression

#### Case 1: cognitively unimpaired at treatment initiation

A cognitively normal, non-Hispanic White woman was enrolled in the study in April 2011, at the age of 63.5 years (born in 1947), and followed for approximately 15 years, through the month 174 visit. The patient completed 18 years of education and had a professional or executive occupational background. The patient had an APOE ϵ3/ϵ3 genotype and reported a maternal history of dementia. No subjective cognitive decline was reported prior to enrollment. There was no history of hypertension or Parkinson’s disease. Donanemab was first documented in September 2025, at the month 174 visit, the last visit with available data; all findings described below therefore precede treatment initiation.

She was assessed on cognition 10 times during the observation period. MMSE scores were 30 at every visit except months 48 and 126, when a single item was missed (MMSE 29), with return to 30 at the following visit (**Figure 1**). The CDR global score was 0 throughout, and the Clinical Dementia Rating–Sum of Boxes (CDR-SB) score was 0 at every visit except month 138, when it was 0.5, returning to 0 thereafter (**Figure 1**). The FAQ score was 0 at every visit except month 12, when it was 2 (**Table 1**). All assessments preceded donanemab initiation at month 174.

She demonstrated a progressive AD biomarker trajectory over 15 years (**Figure 2**). At baseline in 2011, CSF Aβ42 was 1,959 pg/mL with Tau of 305.5 pg/mL and pTau of 24.82 pg/mL, suggesting a relatively borderline or early pathological profile. Based on the Roche Elecsys assay, CSF biomarker cutoffs indicative of AD pathology were Aβ42 <977 pg/mL, total tau >300 pg/mL, and phosphorylated tau181 (pTau181) >27 pg/mL (13). In 2013 (month 24), Aβ42 transiently increased to 2,323 pg/mL whereas Tau and pTau remained mildly elevated at 313.6 and 25.99 pg/mL, respectively. By 2015 (month 48), Tau increased further to 371.5 pg/mL and pTau to 29.91 pg/mL despite Aβ42 remaining relatively stable at 2,250 pg/mL. In 2017, CSF Aβ42 declined substantially to 1,405 pg/mL whereas Tau and pTau remained elevated at 340.1 and 27.74 pg/mL, consistent with clear amyloid positivity and AD pathology. This abnormal biomarker profile persisted in 2019 (Aβ42 = 1,469 pg/mL, Tau = 357.1 pg/mL, pTau = 27.35 pg/mL) and in 2021, with Aβ42 decreasing to 1,274 pg/mL alongside persistently elevated Tau (351.8 pg/mL) and pTau (27.29 pg/mL). In the latest 2024 plasma analysis, pTau217 was elevated at 0.545 with a ratio of 0.0633. Overall, the longitudinal biomarker pattern reflects a transition from an initial borderline state to a sustained A+T+ AD biomarker profile over time.

Amyloid PET imaging scan was available only in 2024 and showed elevated cortical amyloid deposition across regions 1–5. At this same visit, cognitive and functional measures remained normal (MMSE = 30, CDR global = 0, CDR-SB = 0, FAQ = 0). This scan, obtained approximately 1 year before donanemab was initiated, provided the biomarker basis for treatment in a clinically unimpaired individual.

#### Case 2: transition from cognitively normal to MCI at treatment initiation

A non-Hispanic White woman was enrolled in the study in 2018, cognitively normal, at the age of 66.1 years (born in 1952), and followed for approximately 8 years. She had completed 18 years of education, worked as a mid-level professional or small business owner, and retired in 2012. She had an APOE ϵ3/ϵ4 genotype, and both mother and father had dementia. There was no history of hypertension or Parkinson’s disease. Donanemab was first documented on February 21, 2026, immediately before the month 90 visit, at which the diagnosis changed from cognitively normal to MCI; posttreatment follow-up was therefore less than 1 month.

Her clinical diagnosis remained cognitively normal until the month 90 visit in March 2026. Her cognition was assessed five times during the study period. She consistently scored MMSE = 30 until the month 78 visit in February 2025, when she scored 28, returning to 30 at month 90. She scored CDR global=0 and CDR-SB=0 at all visits (**Figure 1**). Despite normal scores from usual cognitive tests, objective memory impairment for age and education (Wechsler Memory Scale 7 Logical Memory II) and subjective memory complaint were reported at the month 90 visit. Caregivers also reported memory complaints, and a physician clinically diagnosed her with MCI with high confidence at month 90, within weeks of donanemab initiation.

She demonstrated a longitudinal biomarker profile consistent with persistent AD-related pathology across both CSF and plasma measures. At baseline in 2018, CSF biomarkers showed markedly reduced Aβ42 (1,129 pg/mL) together with elevated Tau (387 pg/mL) and pTau (35.2 pg/mL), supporting an amyloid-positive and tau-positive profile consistent with underlying AD pathology. In 2019 at the 12m visit, plasma biomarkers further supported this pattern, with pTau217 measured at 0.149 and an Aβ42/Aβ40 ratio of 0.0758. By 2020 at the m24 visit, CSF Aβ42 remained low at 1,058 pg/mL whereas Tau (323.4 pg/mL) and pTau (29.99 pg/mL) remained elevated. In the most recent 2025 plasma assessment at the month 78 visit, pTau217 increased substantially to 0.313 whereas the plasma Aβ42/Aβ40 ratio declined to 0.0621 (**Figure 2**). Amyloid PET imaging at this time confirmed elevated cortical amyloid deposition across regions 1–5. Overall, the longitudinal biomarker trajectory suggests sustained and gradually worsening AD pathology over time, despite relatively preserved cognition during much of the follow-up period.

#### Case 3: progression from MCI to mild dementia after treatment initiation

A non-Hispanic White woman was enrolled in the study in 2019, at the age of 64.5 years (born in 1954), and presented with MCI. The patient reported perceived cognitive decline beginning in 2014, several years prior to study enrollment. The APOE genotype was ϵ3/ϵ3. She had completed 12 years of education, worked in managerial roles, and retired in 2016. Her mother had dementia but father did not. She had a history of hypertension but no history of Parkinson’s disease. Lecanemab was first documented at the month 66 visit (January 2025) and was still recorded at the month 78 visit (December 2025), providing approximately 12 months of posttreatment observation.

She demonstrated persistent MCI across longitudinal follow-up with fluctuating but overall declining MMSE performance. She enrolled with an MMSE score of 28, and at the two most recent assessments, she scored 24 (January 2025, month 66) and 23 (December 2025, month 78), respectively. She demonstrated persistent cognitive impairment across longitudinal CDR assessments, with CDR Global scores fluctuating between 0.5 and 1.0 and CDR-SB scores ranging from 4.0 to 6.0. Although mild temporary improvement was observed at some visits, the overall trajectory suggests progressive cognitive and functional decline over time. At the time of lecanemab initiation (month 66), her CDR global score was 0.5 and CDR-SB 4.0; by month 78, approximately 12 months later, she progressed to CDR global score=1 and CDR-SB=5.0, clinically mild dementia. Throughout the study period, she presented both subjective and objective cognitive impairment, and caregivers also complained about memory impairment (**Figure 1**).

She demonstrated a longitudinal biomarker profile consistent with persistent AD-related pathology across both CSF and plasma measures. At baseline in 2019, CSF biomarkers showed markedly reduced Aβ42 (1,156 pg/mL) together with elevated Tau (277.7 pg/mL) and pTau (27.07 pg/mL), supporting an amyloid-positive and tau-positive profile consistent with underlying AD pathology. Plasma biomarkers obtained at the same visit further supported this pattern, with pTau217 measured at 0.107 and an Aβ42/Aβ40 ratio of 0.0716. During follow-up, plasma pTau217 increased substantially to 0.43 at the month 54 visit in 2024, suggesting progression of tau-related pathology. Although pTau217 levels later declined somewhat at month 66 (0.184) and month 78 (0.187), the plasma Aβ42/Aβ40 ratio progressively decreased to 0.0651 by month 78, remaining consistent with persistent amyloid abnormality (**Figure 2**). At the month 78 visit, approximately 12 months after lecanemab initiation, she continued to demonstrate biomarker evidence of AD pathology together with clinically significant cognitive impairment.

Repeat amyloid PET imaging at 78 months confirmed persistent amyloid elevation, primarily in cortical region 1.

## Discussion

This case series provides longitudinal real-world observations of patients receiving anti-amyloid therapies across three clinically distinct presentations that remain underrepresented in clinical trial evidence. Rather than establishing treatment effectiveness, these cases illustrate how anti-amyloid therapies are being implemented in routine clinical practice and highlight unresolved challenges in biomarker interpretation, clinical staging, longitudinal monitoring, and treatment response that warrant investigation in larger real-world cohorts. Specifically, the three cases demonstrate treatment initiation in a cognitively unimpaired but biomarker-positive individual, detection of early cognitive decline despite preserved global cognitive screening, and continued disease progression despite anti-amyloid therapy. These findings suggest that anti-amyloid therapies are increasingly being used outside traditional trial-defined symptomatic populations and may be initiated during prolonged preclinical phases of AD. This emerging treatment landscape reflects concerns raised by the International Working Group (IWG), which cautioned that expanding biomarker-based definitions of AD may increase pressure to initiate anti-amyloid therapy in cognitively normal individuals, including off-label treatment (14). The present case series provides real-world examples of these evolving clinical scenarios. These findings further underscore that clinical presentation and biomarker status do not uniformly align at the time of treatment initiation, particularly among individuals treated outside trial-defined symptom thresholds.

A notable finding was the dissociation between biomarker abnormalities and overt cognitive decline in cases 1 and 2. Both individuals demonstrated sustained A+T+ biomarker profiles over several years while maintaining largely preserved MMSE, CDR, and functional performance. In particular, case 1 remained cognitively and functionally normal for approximately 15 years despite progressive amyloid and tau abnormalities, elevated plasma pTau217, and amyloid PET positivity. Case 1 also highlights the ongoing debate regarding the classification and treatment of cognitively normal, biomarker-positive individuals. Under the 2024 IWG framework, this participant would be considered an “asymptomatic at-risk” individual rather than having AD, whereas under the 2024 Alzheimer’s Association (AA) criteria, the same participant would meet criteria for stage 1 AD based on biomarker evidence alone (14, 15).

The classification of such individuals remains a matter of debate. Under the 2024 AA criteria, abnormal core biomarkers are diagnostic of AD irrespective of symptoms, so a cognitively unimpaired person with amyloid and tau positivity has stage 1 disease. A substantial proportion of amyloid-positive, cognitively unimpaired individuals will not develop dementia within their remaining lifetime, and progression risk varies markedly with age, APOE genotype, and tau status (16). The disagreement has practical consequences, because a biological diagnosis in the absence of symptoms supplies a rationale for treating a person who is functioning normally, as in case 1. We therefore use preclinical AD descriptively, to denote biomarker positivity without cognitive impairment, rather than as a disease designation.

Similarly, case 2 demonstrated persistent biomarker abnormalities with only subtle cognitive transition emerging later through subjective complaints and memory-specific impairment despite preserved global cognitive scores. These findings support the concept that pathological AD-related changes may precede clinically detectable impairment by many years and highlight the potential role of cognitive reserve in delaying overt symptom manifestation (17). Both participants had relatively high educational attainment and professional occupational backgrounds, factors previously associated with cognitive reserve and resilience to clinical expression of AD pathology (17, 18).

In contrast, case 3 demonstrated a more symptomatic trajectory characterized by persistent MCI and progression toward mild dementia despite anti-amyloid therapy exposure. Unlike preclinical cases, this patient exhibited sustained subjective and objective cognitive impairment throughout follow-up, declining MMSE performance, worsening CDR measures, and persistent biomarker abnormalities. Progression continued over the approximately 12 months following lecanemab initiation. Given the short exposure and the decline expected in untreated MCI due to AD over the same interval, this cannot be interpreted as treatment failure, although it illustrates that clinically meaningful decline may continue during the first year of therapy. Together, these cases illustrate the substantial heterogeneity that exists among real-world recipients of anti-amyloid therapies and emphasize that biomarker positivity alone may not correspond uniformly to immediate clinical decline.

A separate clinical consideration involves the safety implications of initiating anti-amyloid therapies in biomarker-positive individuals who remain minimally symptomatic or cognitively normal, particularly among APOE ϵ4 carriers. APOE ϵ4 status has consistently been associated with increased risk of amyloid-related imaging abnormalities (ARIA), including both edema and microhemorrhage, during treatment with anti-amyloid monoclonal antibodies (5, 6, 19). This issue is particularly relevant in the present case series, as case 2, who carried an APOE ϵ3/ϵ4 genotype, received donanemab during a very early transitional stage despite preserved global cognitive functioning. Reflecting these concerns, recent European appropriate use recommendations have adopted more conservative eligibility approaches and do not recommend treatment for APOE ϵ4 homozygous carriers because of elevated ARIA risk (20, 21). Nevertheless, both lecanemab and donanemab were approved for clinical use regardless of APOE ϵ4 carrier status, highlighting the ongoing tension between broader treatment access and individualized risk stratification in real-world practice.

Case 1 also warrants comment because donanemab was initiated well outside currently recommended treatment boundaries. Both agents are labeled for, and appropriate use recommendations restrict treatment to, patients with MCI or mild dementia due to AD, operationally objective cognitive impairment with a CDR global score of 0.5 to 1, and confirmed amyloid positivity (5, 6). At initiation, this participant had an MMSE of 30, a CDR global score of 0, CDR-SB of 0, and FAQ of 0, sustained across 15 years of follow-up; she met neither the labeled indication nor appropriate use criteria on clinical grounds, despite unambiguous amyloid and tau positivity. Efficacy in cognitively unimpaired, amyloid-positive individuals has not been established and is the question ongoing clinical trials are designed to answer. In the absence of demonstrated benefit, the recognized risks of therapy, including ARIA and the burden of repeated MRI surveillance, are incurred without corresponding expected clinical gain. Real-world analyses indicate that many treated patients fall outside trial criteria, but treatment at CDR 0 represents a categorically different departure, and how such use should be discussed during consent, documented, and monitored is not addressed by current frameworks.

This study also highlights the limitations of relying exclusively on global cognitive screening tools such as MMSE or CDR in highly educated individuals with substantial cognitive reserves. In case 2, MMSE and CDR scores remained within normal ranges despite progressive objective memory impairment detected through age- and education-adjusted Wechsler Logical Memory testing, which ultimately captured the transition from cognitively normal status to MCI. These findings suggest that standard global screening instruments, which do not adequately account for cognitive reserve factors, may underestimate early cognitive decline in highly educated individuals.

These findings have important implications for the identification of treatment-eligible patients in clinical practice. Recent commentaries have emphasized that inaccurate clinical staging may lead to inappropriate biomarker testing and overestimation of dementia risk and ultimately influence treatment decisions, underscoring the importance of integrating comprehensive neuropsychological assessment with biomarker evaluation when determining treatment eligibility (16). Current anti-amyloid therapy eligibility criteria and clinical trial frameworks rely heavily on global cognitive screening measures, which may contribute to challenges in identifying appropriate candidates and to the relatively low treatment uptake observed in real-world settings (22, 23). Donanemab trials primarily utilized MMSE-based thresholds that were not adjusted for age or educational attainment, whereas lecanemab trials incorporated Wechsler Logical Memory measures adjusted for age but not for broader cognitive reserve factors such as educational background (5, 6). As a result, subtle but clinically meaningful decline in highly educated individuals with substantial cognitive reserve may remain undetected despite ongoing underlying disease progression. Rather than broadly expanding eligibility to preclinical populations, the present findings suggest the importance of refining clinical assessment approaches to better account for cognitive reserve factors and memory-specific impairment. This issue may become increasingly relevant as ongoing trials, including studies evaluating donanemab in preclinical AD populations (24), continue to explore treatment initiation at earlier stages of disease progression.

### Limitations

Several limitations should be considered when interpreting these findings. First, this study is based on a small case series drawn from ADNI participants; therefore, the findings should be interpreted cautiously and may not be generalizable to broader clinical populations receiving anti-amyloid therapies in routine practice. Second, interpreted amyloid PET scan results became available only after cohort 4 data collection beginning in 2023, limiting the ability to evaluate longitudinal changes in amyloid burden over time. Third, biomarker data were collected across different ADNI phases using both CSF and plasma assays, which may introduce methodological variability and limit direct longitudinal comparability across visits. Fourth, posttreatment follow-up was minimal: Case 1 had no observations after donanemab initiation, case 2 less than 1 month, and only case 3 approximately 12 months. The trajectories reported are therefore predominantly pretreatment and support no conclusion, favorable or unfavorable, regarding treatment effectiveness or disease modification. Finally, as an observational case series, causal inferences regarding treatment effects cannot be established.

These limitations notwithstanding, this study illustrates variability in clinical trajectories among biomarker-positive individuals receiving anti-amyloid therapy in a real-world observational context. The observed differences across cases underscore the importance of longitudinal multimodal assessment when interpreting treatment courses and disease progression in AD.

### Future directions

Future work should examine larger, longitudinal cohorts to better describe variation in clinical trajectories across treatment stages, with particular attention to differences between biomarker status and observable cognitive change at treatment initiation. Studies should also evaluate how APOE ϵ4 status, baseline biomarker burden, and sensitive memory-specific assessments can inform patient selection and monitoring. These efforts may help refine eligibility criteria and support more individualized treatment decisions as anti-amyloid therapies are increasingly considered earlier in the AD continuum.

## Conclusion

This case series describes three biomarker-positive individuals from ADNI who were exposed to anti-amyloid monoclonal antibody therapy and followed longitudinally with clinical, cognitive, functional, fluid biomarker, and amyloid PET assessments. Representing three distinct clinical scenarios across the AD continuum, the cases demonstrate substantial heterogeneity in clinical trajectories. The cases also suggest that reliance on global cognitive screening alone may underestimate early cognitive changes in biomarker-positive individuals. While some individuals exhibited prolonged preservation of cognition despite persistent biomarker evidence of AD pathology, others demonstrated progressive cognitive decline despite treatment exposure. These observations highlight the complexity of the relationship between biomarker abnormalities and clinical presentation and illustrate the challenges of implementing anti-amyloid therapies beyond traditional symptomatic trial populations. Larger real-world observational studies with longer follow-up are needed to better characterize clinical outcomes and biomarker trajectories among patients receiving anti-amyloid therapies.

## Data Availability

Publicly available datasets were analyzed in this study. This data can be found here: https://adni.loni.usc.edu/data-samples/adni-data/.

## References

[1] 2024 Alzheimer’s disease facts and figures. Alzheimer’s Dementia. (2024) 20:3708–821. doi: 10.1002/ALZ.13809 38689398 PMC11095490

[2] Rajan KB Weuve J Barnes LL McAninch EA Wilson RS Evans DA . Population estimate of people with clinical Alzheimer’s disease and mild cognitive impairment in the United States (2020–2060). Alzheimer’s Dementia. (2021) 17:1966–75. doi: 10.1002/ALZ.12362 34043283 PMC9013315

[3] Villemagne VL Burnham S Bourgeat P Brown B Ellis KA Salvado O . Amyloid β deposition, neurodegeneration, and cognitive decline in sporadic Alzheimer’s disease: a prospective cohort study. Lancet Neurol. (2013) 12:357–67. doi: 10.1016/S1474-4422(13)70044-9 23477989

[4] Porsteinsson AP Isaacson RS Knox S Sabbagh MN Rubino I . Diagnosis of early Alzheimer’s disease: Clinical practice in 2021. J Prev Alzheimers Dis. (2021) 8:371–86. doi: 10.14283/JPAD.2021.23 34101796 PMC12280795

[5] Sims JR Zimmer JA Evans CD Lu M Ardayfio P Sparks J . Donanemab in early symptomatic Alzheimer disease: The TRAILBLAZER-ALZ 2 randomized clinical trial. JAMA. (2023) 330:512–27. doi: 10.1001/JAMA.2023.13239 37459141 PMC10352931

[6] van Dyck CH Swanson CJ Aisen P Bateman RJ Chen C Gee M . Lecanemab in early Alzheimer’s disease. N Engl J Med. (2023) 388:9–21. doi: 10.1056/NEJMoa2212948 36449413

[7] Manly JJ Deters KD . Donanemab for Alzheimer disease—Who benefits and who is harmed? JAMA. (2023) 330:510–1. doi: 10.1001/JAMA.2023.11704 37459138

[8] Jia J Ning Y Chen M Wang S Yang H Li F . Biomarker changes during 20 years preceding Alzheimer’s disease. N Engl J Med. (2024) 390:712–22. doi: 10.1056/NEJMOA2310168 38381674

[9] Sabbagh MN Zhao C Mahendran M Jang SR Laliberté F Toyosaki H . Characterizing the journey of early Alzheimer’s disease in patients initiating lecanemab treatment in the United States: A real-world evidence study. (2025) 14:1115–27. doi: 10.1007/s40120-025-00756-4 PMC12089008 40319433

[10] Brixner DI Zhao C Toyosaki H Frech FH Rosenbloom MH . Initial real-world evidence for lecanemab in the United States. (2026) 43:69–76. doi: 10.1007/s40266-025-01261-x PMC12855276 41160347

[11] Bregman N Nathan T Shir D Omer N Levy MH David AB . Lecanemab in clinical practice: real-world outcomes in early Alzheimer’s disease. Alzheimers Res Ther. (2025) 17:119. doi: 10.1186/S13195-025-01763-1 40437535 PMC12117801

[12] Alzheimer’s Disease Neuroimaging Initiative: ADNI . Available online at: https://adni.loni.usc.edu/data-samples/adni-data/ (Accessed July 31, 2026).

[13] Blennow K Shaw LM Stomrud E Mattsson N Toledo JB Buck K . Predicting clinical decline and conversion to Alzheimer’s disease or dementia using novel Elecsys Aβ(1-42), pTau and tTau CSF immunoassays. Sci Rep. (2019) 9:19024. doi: 10.1038/S41598-019-54204-Z 31836810 PMC6911086

[14] Dubois B Villain N Schneider L Fox N Campbell N Galasko D . Alzheimer disease as a clinical-biological construct—An international working group recommendation. JAMA Neurol. (2024) 81:1304–11. doi: 10.1001/JAMANEUROL.2024.3770 39483064 PMC12010406

[15] Jack CR Andrews JS Beach TG Buracchio T Dunn B Graf A . Revised criteria for diagnosis and staging of Alzheimer’s disease: Alzheimer’s Association Workgroup. Alzheimer’s Dementia. (2024) 20:5143–69. doi: 10.1002/ALZ.13859 38934362 PMC11350039

[16] Kiselica AM Gaynor LS Atkins KJ . IWG and AA criteria-Where the differences matter. JAMA Neurol. (2025) 82:628. doi: 10.1001/JAMANEUROL.2025.0775 40257779

[17] Stern Y . Cognitive reserve in ageing and Alzheimer’s disease. Lancet Neurol. (2012) 11:1006. doi: 10.1016/S1474-4422(12)70191-6 23079557 PMC3507991

[18] Corbo I Marselli G Di Ciero V Casagrande M . The protective role of cognitive reserve in mild cognitive impairment: A systematic review. J Clin Med. (2023) 12:1759. doi: 10.3390/JCM12051759 36902545 PMC10002518

[19] Salloway S Chalkias S Barkhof F Burkett P Barakos J Purcell D . Amyloid-related imaging abnormalities in 2 phase 3 studies evaluating aducanumab in patients with early Alzheimer disease. JAMA Neurol. (2022) 79:13–21. doi: 10.1001/JAMANEUROL.2021.4161 34807243 PMC8609465

[20] Cummings J Apostolova L Rabinovici GD Atri A Aisen P Greenberg S . Lecanemab: Appropriate use recommendations. J Prev Alzheimer’s Dis. (2023) 10:362–77. doi: 10.14283/jpad.2023.30 37357276 PMC10313141

[21] Rabinovici GD Selkoe DJ Schindler SE Aisen P Apostolova LG Atri A . Donanemab: Appropriate use recommendations. J Prev Alzheimers Dis. (2025) 12:100150. doi: 10.1016/j.tjpad.2025.100150 40155270 PMC12180672

[22] Urso D Introna A Gnoni V Giugno A Vilella D Zecca C . Donanemab eligibility in early Alzheimer’s disease: A real-world study. J Alzheimer’s Dis. (2025) 105:745–50. doi: 10.1177/13872877251331243 40313052

[23] Rosenberg A Solomon A Bonnard A Daniilidou M Hagman G Hall A . Preparing for the implementation of anti-amyloid therapies in Europe: Assessing real-world eligibility for lecanemab and donanemab in a Swedish memory clinic. J Prev Alzheimer’s Dis. (2026) 13:100476. doi: 10.1016/j.tjpad.2025.100476 41544372 PMC12835597

[24] Yaari R Holdridge KC Williamson M Wessels AM Shcherbinin S Kotari V . Donanemab in preclinical Alzheimer’s disease: Screening and baseline data from TRAILBLAZER‐ALZ 3. (2025) 21:e70662. doi: 10.1002/alz.70662 PMC12439032 40955720

